# MXene-Based Gas Sensors for NH_3_ Detection: Recent Developments and Applications

**DOI:** 10.3390/mi16070820

**Published:** 2025-07-17

**Authors:** Yiyang Xu, Yinglin Wang, Zhaohui Lei, Chen Wang, Xiangli Meng, Pengfei Cheng

**Affiliations:** School of Aerospace Science and Technology, Xidian University, Xi’an 710126, China; xyy13243996167@163.com (Y.X.); lzhcatgod@163.com (Z.L.); wangchen01@xidian.edu.cn (C.W.); xlmeng811908@163.com (X.M.)

**Keywords:** MXene, ammonia, gas sensing mechanism, heterojunction engineering

## Abstract

Ammonia, as a toxic and corrosive gas, is widely present in industrial emissions, agricultural activities, and disease biomarkers. Detecting ammonia is of vital importance to environmental safety and human health. Sensors based on MXene have become an effective means for detecting ammonia gas due to their unique hierarchical structure, adjustable surface chemical properties, and excellent electrical conductivity. This study reviews the latest progress in the use of MXene and its composites for the low-temperature detection of ammonia gas. The strategies for designing MXene composites, including heterojunction engineering, surface functionalization, and active sites, are introduced, and their roles in improving sensing performance are clarified. These methods have significantly improved the ability to detect ammonia, offering high selectivity, rapid responses, and ultra-low detection limits within the low-temperature range. Successful applications in fields such as industrial safety, food quality monitoring, medical diagnosis, and agricultural management have demonstrated the multi-functionality of this technology in complex scenarios. The challenges related to the material’s oxidation resistance, humidity interference, and cross-sensitivity are also discussed. This study aims to briefly describe the reasonable design based on MXene sensors, aiming to achieve real-time and energy-saving environmental and health monitoring networks in the future.

## 1. Introduction

Ammonia (NH_3_), a crucial chemical raw material and potential clean energy carrier, plays a central role in modern industrial and agricultural production as well as in daily life. However, its extensive application also comes with strict requirements for environmental, health, and safety monitoring, which has driven the development of high-performance, low-cost ammonia gas sensors. In ammonia synthesis plants, fertilizer production, refrigeration systems [1], and chemical processes, ammonia is both a key raw material and a potential source of hazardous leaks. Even at lower concentrations (such as 25–50 ppm), ammonia can cause severe irritation (affecting eyes and the respiratory system) [2], while high concentrations of ammonia gas pose the risk of fires and explosions. Therefore, real-time, in situ, and low-power leakage monitoring sensors are crucial for ensuring industrial safety and optimizing production processes. Ammonia is an efficient refrigerant widely used in large cold storage facilities, food processing plants, and cold chain logistics, and is also one of the gas components that can cause food spoilage. However, leakage in refrigeration systems can directly pollute food or indirectly affect food quality and safety (such as temperature, leading to spoilage) [3]. Strict monitoring of ammonia concentrations in cold storage areas, transportation vehicles, and supermarket refrigerators is essential for preventing food contamination and spoilage, ensuring consumer health, reducing economic losses, and preserving brand reputation. Ammonia is one of the main atmospheric pollutants generated in livestock farming (poultry houses) and fertilizer application processes, and is also an important precursor to PM2.5 [4]. Monitoring ammonia concentrations in farms and their surrounding environments is urgently needed for assessing environmental pollution, optimizing breeding management, achieving precise fertilization, and meeting increasingly strict emission regulations. Among patients with chronic kidney disease, renal failure can lead to ammonia metabolism disorders, resulting in an increase in ammonia content in the blood. To maintain acid–base balance, the human body attempts to excrete excess ammonia through other pathways (such as the lungs), resulting in a significant increase in ammonia concentration in exhaled gas [5]. Studies have shown that the exhaled ammonia concentration in healthy people ranges from 0.4 to 0.9 ppm, while patients with chronic kidney disease experience increases from 1.5 to 3 ppm or even higher, especially when the condition worsens or the demand for dialysis increases [6]. This indicates that the ammonia concentration in human exhaled gas is closely related to the degree of renal function impairment and the progression status of the disease. In the field of non-invasive medical diagnosis, ammonia in exhaled gas has become an important potential biomarker for evaluating kidney function and monitoring the progression of kidney diseases [7]. However, there are still technical challenges regarding breath analysis, including the decrease in sensitivity caused by humidity (>90% RH), cross-interference of coexisting gases (ethanol and CO_2_), and sub-ppm detection requirements. Gas sensors demonstrate unique advantages in breath analysis compared to conventional techniques like mass spectrometry or gas chromatography, offering portability, real-time monitoring capability, and cost-effectiveness for point-of-care diagnostics.

Among various sensing architectures, chemiresistive gas sensors stand out due to their inherent compatibility with miniaturized systems, low power consumption, and tunable sensitivity through the use of nanostructured materials engineering. By leveraging functional nanomaterials (e.g., metal oxide semiconductors [8], conductive polymers [9], and 2D materials [10]), these sensors achieve direct electrical signal transduction upon gas adsorption, enabling rapid response and sub-ppm detection thresholds without complex sample pretreatment. Their scalable fabrication processes further support integration into wearable devices or IoT-enabled platforms, positioning chemiresistive sensors as a transformative solution for decentralized metabolic monitoring and personalized healthcare management.

MXenes are a class of two-dimensional layered materials with the general chemical formula M_n+1_X_n_T_x_ (M: transition metal; X: C/N; T_x_: surface termini). Their unique microstructure results from the selective etching of the “A” atomic layer in the precursor MAX phase, forming atomic-thick layers composed of strongly bonded M-X units [11]. The exposed M atoms are saturated with terminal groups such as –O, –OH, and –F, and the type of terminal (–T_x_) directly regulates the electronic structure (work function/gap), hydrophilicity, and chemical activity. The interlayer distance of MXene layers can be dynamically adjusted through intercalation or swelling, directly affecting the specific surface area and gas diffusion channels. The vacancies, edge defects, etc., introduced during the etching process can serve as high-activity adsorption centers. MXene materials (such as Ti_3_C_2_T_x_) offer revolutionary solutions for gas detection at room temperature with their unique two-dimensional layered structure, adjustable surface functional groups (–O, –OH, –F), and ultra-high electrical conductivity [12]. Compared to the limitations of traditional metal oxide sensors, which rely on high-temperature activation, the rich surface active sites of MXene can achieve efficient adsorption and charge transfer of target gas molecules at room temperature, significantly reducing power consumption and avoiding thermal degradation problems [13]. The controllable interlayer nanochannels and high specific surface area further enhance the gas diffusion kinetics and interfacial reactivity, laying the foundation for the rapid capture and identification of trace ammonia [14]. These characteristics make MXene-based sensors an ideal candidate material for achieving high-precision and low-power breath analyzers.

In recent years, significant progress has been made in the research of MXene-based room-temperature gas sensors for ammonia detection. Zahra et al. [15] developed a highly recyclable and stable nitrogen-doped Ti_3_C_2_T_x_ sensor for ammonia detection at room temperature, the response of which was 3.7% to 100 ppm ammonia gas. Lee et al. [16] studied the room-temperature gas sensitivity performance of Ti_3_C_2_T_x_ nanosheets, showing that the Ti_3_C_2_T_x_ sensor had the highest sensitivity to ammonia at room temperature. Theoretically, the detection limit of ammonia is approximately 9.27 ppm, which is a better performance compared to the other two-dimensional material sensors. In addition, the hydrophobic property (contact angle >100°) and functional modifications (such as polydopamine coatings) of MXene significantly suppress performance degradation under high humidities (90% RH) (response fluctuation <15%). At the same time, its low power consumption meets the application requirements of wearable devices [17]. However, the practical application of MXene-based sensors is still limited by core challenges such as insufficient environmental stabilities, cross-interference in complex exhalation matrices, and limited detection ranges.

Two-dimensional transition metal carbides/nitrides (MXenes) have shown great potential in the field of gas sensing, especially in detecting ammonia gas (NH_3_) at room temperature, drawing extensive research attention. Although numerous studies have reported NH_3_ sensors based on MXenes, this study aims to provide a unique perspective and a systematic summary, strictly focusing on MXene-based NH_3_ sensors that operate at room temperature. It not only systematically reviews their performance indicators (sensitivity, detection limit, response/recovery time, and selectivity) but also deeply analyzes the key factors limiting their performance at room temperature and evaluates and classifies the effectiveness and potential mechanisms of the performance optimization strategies proposed in the current literature. This will provide clear ideas for the rational design of future high-performance room-temperature devices. This study not only focuses on high performances in the laboratory environment but also emphasizes the importance of a combined high performance and high stability in complex practical applications (such as environments with humidity fluctuations and coexisting gas interference and when in long-term operation). It analyzes the performance of existing sensors under these challenging scenarios and highlights the development of new device architectures and material design concepts to address these issues. Based on a comprehensive review and critical analysis of existing research, this study presents unique insights into the future development directions of MXene-based room-temperature NH_3_ sensors, clearly identifying the current research gaps and key scientific problems that need to be addressed.

## 2. Sensitivity Mechanism of the Gas Sensor

The surface-terminating functional groups (–O, –OH, and –F) of MXene materials endow them with unique physicochemical properties, highlighting their significant potential in the field of ammonia gas sensing. Lee et al. [16] found that when a Ti_3_C_2_T_x_ MXene sensor was exposed to ethanol, methanol, acetone, and ammonia (100 ppm), it exhibited P-type sensing behavior, meaning the film resistance increased and then recovered after being separated from the gas. The response to ammonia was the highest, and that to acetone was the lowest. Theoretically, Ti_3_C_2_T_x_ has metallic properties, but its surface functional groups (such as –O, –OH) can induce surface dipole polarization, reducing the electrical conductivity to the semiconductor level and forming a narrow bandgap semiconductor. This P-type characteristic may originate from the H_2_O and O_2_ molecules introduced during the preparation process (Al etching), which act as P-type dopants to regulate the carrier concentration [18,19].

The room-temperature NH_3_ sensitivity mechanism of Ti_3_C_2_T_x_ MXene can be classified into two mechanisms:

### 2.1. Adsorbed Oxygen Model

When the sensor is exposed to the air environment, O_2_ molecules in the air adsorb onto the surface of the sensor, forming different types of oxygen (O^2−^, O^−,^ and O_2_^−^) [20]. Professor N. Barsan pointed out that the type of oxygen anion is related to the working temperature of the sensor [21]. The possible reaction equation of oxygen on the surface of gas-sensitive materials is as follows:

O_2(gas)_ → O_2(ads)_ (1)

O_2(ads)_ + e^−^ → O_2_^−^_(ads)_ (T < 150 °C) (2)

O_2_^−^_(ads)_ + e^−^ → 2O^−^_(ads)_ (150 °C < T < 300 °C) (3)

O^−^_(ads)_ + 2e^−^ → O^2−^_(ads)_ (T > 300 °C) (4)

**Figure 1:**
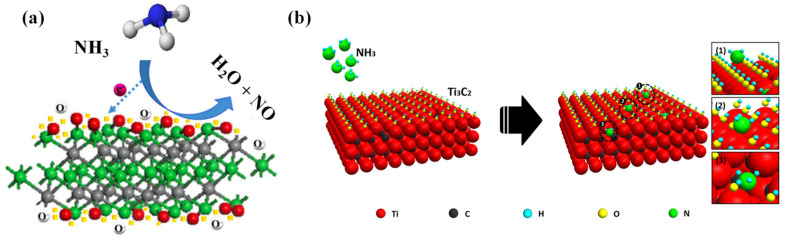
(**a**) The REDOX reaction that occurs between ammonia and the surface of materials; (**b**) a schematic illustration of the possible gas-sensing mechanisms of the Ti_3_C_2_T_x_ for NH_3_ gas [15].

When exposed to RT air, oxygen molecules directly capture electrons in the form of O_2_^−^ on the surface of the gas-sensitive material and form a space charge region on the surface of the gas-sensitive material (Figure 1b). This area can serve as an active site for ammonia adsorption. When ammonia is present on gas-sensitive materials, ammonia molecules undergo REDOX reactions with O_2_^−^, and the free electrons released by the reaction return to the conduction band of the sensitive material [22,23]. This process causes changes in the carrier concentration of the material, resulting in an increase in resistance (the P-type universal response characteristic of Ti_3_C_2_T_x_). The common formula for explaining the reaction of ammonia with oxygen under RT is as follows [24] (Figure 1a):

4NH_3_ + 5O_2_^−^ → 4NO + 6H_2_O + 5e^−^ (5)

### 2.2. Terminal Functional Groups React Directly

The NH_3_ molecule reacts with the oxygen-containing functional groups (–O/–OH) on the surface of Ti_3_C_2_T_x_. Lee et al. [15] first proposed Ti_3_C_2_T_x_, which accounts for most of the interactions between the carrier and the gas sensing mechanism. For MXenes, gas adsorption can occur at active defect sites on the surface of Ti_3_C_2_T_x_, or it can be the result of interaction with surface functional groups. For functional groups, gas absorption is caused by dispersing forces such as electrostatic forces. Due to the weak intermolecular forces, the resistance change is relatively small. On the other hand, the substitution of surface functional groups by gas molecules may lead to gas absorption, resulting in carrier transfer between the adsorbent and the adsorbate gas and causing a significant change in the resistance of the Ti_3_C_2_T_x_ film. Research suggests that the Ti_3_C_2_T_x_ sensor’s sensing mechanisms, including defects and functional groups, influence the absorption of the target gas. The target gases bind to the structural defects of Ti_3_C_2_T_x_ nanosheets, while others are bound to the surface ends, such as –O and –OH, and interact with each other. Adsorbed gases (such as ethanol, methanol, acetone, and ammonia) can all be used as electron donor gases, and the Ti_3_C_2_T_x_ film exhibits P-type sensing behavior for all four gases. At the active sites of Ti_3_C_2_T_x_ nanosheets, electron donor molecules are mainly absorbed through the dispersion forces between polarized gas molecules and some charged functional groups or defects. If the adsorbed gas molecules are absorbed by functional groups such as hydroxyl groups, the bonding between them through hydrogen bonds will be stronger, and the binding energy will also be greater [25]. Therefore, electrons can be transferred from the adsorbed gas to Ti_3_C_2_T_x_, resulting in a decrease in the majority carrier concentration of the Ti_3_C_2_T_x_ film and an increase in the resistance of the Ti_3_C_2_T_x_ device. As shown in Figure 1, depending on the type of surface termination, there may be two possible reactions for the electron transfer process of the Ti_3_C_2_T_x_ film to NH_3_ gas. The reaction pathways of the chemical substances on the surface of Ti_3_C_2_T_x_ with NH_3_ are –O in Equation (6) [26] and –OH in Equation (7) [27].

2NH_3_ + 3O^−^ → N_2_ + 3H_2_O +3e^−^ (6)

NH_3_ + OH^−^ → NH_2_ + H_2_O +e^−^ (7)

## 3. Optimizing the Performance of MXene for NH_3_

Despite having particular ammonia-active sites and a surface rich in functional groups (–O, –F, and –OH), the practical application of MXene has been severely hindered by the inherent limitations of the material. (1) MXene nanosheet stacking: the layers will be drawn near one another by the van der Waals forces between MXene, which will reduce the material’s interlayer spacing and prevent gas diffusion [28]. (2) When exposed to oxidizing environments (e.g., strong oxidants, elevated temperature, and light/ultraviolet radiation), the low-valence Ti species (Ti^3+^ and Ti^2+^) present on MXene surfaces and edges undergo oxidation to the more stable Ti^4+^ state. This oxidation process diminishes the material’s electrical conductivity [29]. Concurrently, the original edge structure is compromised, and surface functional groups (e.g., –OH, –F, and –O) are either desorbed, replaced, or transformed. Consequently, active sites essential for target gas adsorption are lost, leading to poor stability in MXene-based sensors. The oxidation reaction triggered by oxygen and water can be represented by Equations (8)–(11) [30,31,32]. (3) Strong polar groups, such as –OH, –O, and –F, on the surface of MXene are prone to form hydrogen bonds with water molecules, significantly reducing the adsorption sites for target gases in high-humidity environments. Therefore, in high-humidity environments, the performance of MXene sensors is greatly reduced and their moisture resistance is insufficient. These issues result in the poor anti-interference ability of MXene sensors, which poses a barrier to their practical application.

Ti_3_C_2_O_2(s)_ + 3O_2(g)_ → 3TiO_2(s)_ + C_(s)_ + CO_2(g)_ (8)

Ti_3_C_2_O_2(s)_ + 4H_2_O → 3TiO_2(s)_ + 2C_(s)_ + 4H_2(g)_ (9)

2Ti_3_C_2_(OH)_2(s)_ + 9O_2(g)_ → 6TiO_2(s)_ + 4CO_2(g)_ + 2H_2_O (10)

2Ti_3_C_2_(OH)_2(s)_ + 11H_2_O → 6TiO_2(s)_ + CO_(g)_ + CO_2(g)_ + 2CH_4(g)_ +9H_2(g)_ (11)

To overcome these limitations and enhance the overall performance of MXene-based ammonia sensors, researchers have developed material engineering strategies focused on the following approaches. 1. Incorporating metal oxides, transition metal sulfides, or similar materials between MXene layers or onto their surfaces. These materials act as intercalants, expanding the interlayer spacing to expose additional active sites and facilitate gas diffusion. They form heterojunctions with MXene to modulate charge transfer. Simultaneously, they serve as protective barriers against MXene oxidation and enhanced material stability. 2. Formed polymer coatings on the MXene surface. These coatings provide dual functionality: they shield MXene from oxidation and create a hydrophobic layer that repels water molecules. This significantly improved the material stability and sensing performance in high-humidity environments. 3. Precious metals can act as physical barriers to isolate the active sites (Ti^3+^) at the edge of MXene from O_2_/H_2_O, thereby delaying the oxidation process. Precious metals can also stabilize the surface charge of MXene and reduce signal fluctuations caused by humidity changes.

The following sections provide a detailed account of these optimization strategies, highlighting how they enhance NH_3_ sensing performance and address the dual challenges of antioxidant and moisture resistance.

### 3.1. The Introduction of Metal Oxides

Metal oxides optimize the gas-sensitive performance of MXene through the dual effects of increasing the intercalation distance and surface passivation. Their nanoparticles are embedded between MXene layers as intercalating agents, effectively overcoming the layer stacking caused by van der Waals forces and fully exposing the masked functional groups (–O/–F/–OH) and active sites. In addition, the metal oxide coating inhibits the oxidation erosion of the Ti^3+^ active sites at the edge of MXene by H_2_O/O_2_ in the environment through physical barrier and oxygen vacancy quenching mechanisms. This structural regulation increases the contact surface area of NH_3_ molecules, endowing the material with long-term stability. In contrast, the metal oxide surface has a large number of oxygen vacancies and Lewis acidic sites (metal cation defects), which can lead to strong chemical adsorption of alkaline ammonia molecules through hydrogen bonding and acid–base interactions, enhancing gas adsorption and reaction efficiency. Metal oxides can form p-n heterojunctions within the MXene interface, altering the Fermi level height. When ammonia molecules interact with the composite material, electron transfer is accelerated, and MXene, as a conductive channel, further amplifies the signal output. Under the synergistic effect of the two methods, highly sensitive ammonia detection was ultimately achieved at room temperature.

**Figure 2:**
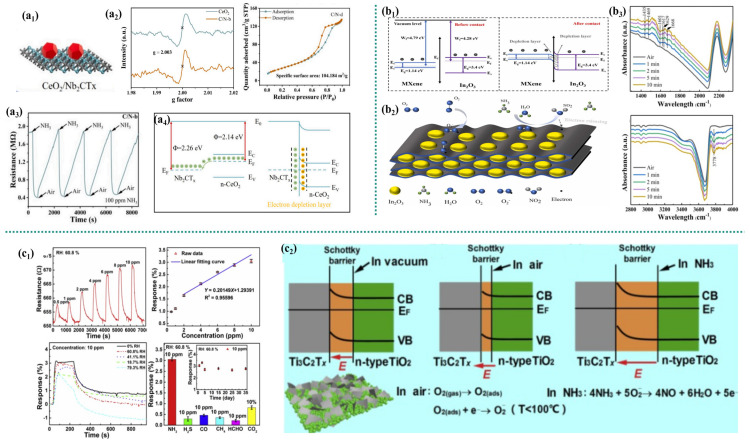
(**a**) Nb_2_CTx MXene with CeO_2_ nanoparticles [33]: (**a_1_**) schematic diagram, (**a_2_**) N_2_ adsorption–desorption curve, (**a_3_**) four consecutive response-recovery curves and (**a_4_**); (**b**) In_2_O_3_ nanowires into Ti_3_C_2_Tx MXene [36]: (**b_1_**) schematic of the device structure and energy levels, (**b_2_**) schematic of the gas sensing mechanism, and (**b_3_**) absorbance spectra for NH_3_ at different exposure times; (**c**) Ti_3_C_2_T_x_/TiO_2_ composites [38]: (**c_1_**) the dynamic response recovery curves of the gas sensor to different concentrations of ammonia gas, the fitted curves, the response recovery curves under different humidity conditions, and the selectivity, and (**c_2_**) schematic diagram.

Wang et al. [33] designed and demonstrated a room-temperature NH_3_ gas sensor based on CeO_2_ nanoparticle-functionalized Nb_2_CT_x_ MXene (CeO_2_/Nb_2_CT_x_). Compared with pure CeO_2_, the response of this composite material to NH_3_ has significantly increased fourfold. In addition, the sensor also demonstrates low detection limits, excellent repeatability, and long-term stability, as well as rapid response and recovery times (Figure 2a_3_). In this study, CeO_2_/Nb_2_CT_x_ composites were synthesized using the hydrothermal method (Figure 2a_1_). During this process, Nb_2_CT_x_, serving as the substrate, adsorbs Ce^3+^ ions, promoting their reaction, nucleation, and the growth of CeO_2_ nanoparticles. The large specific surface area of Nb_2_CT_x_ not only provides a growth template for CeO_2_, but also the CeO_2_ nanoparticles effectively act as spacers, inhibiting the re-accumulation of the Nb_2_CT layer, reducing the area of MXene directly exposed to environmental oxygen, suppressing its oxidation, thereby generating more NH_3_ adsorption active sites and increasing the adsorption capacity of the target gas (Figure 2a_2_). Nb_2_CT_x_ mainly acts as a support layer, providing an efficient channel for electron transport at room temperature (RT). CeO_2_ nanoparticles mainly provide the active sites for NH_3_ adsorption. The unique layered structure of the composite material significantly increases the specific surface area, greatly promoting the adsorption and diffusion capacity of NH_3_ molecules. The introduction of Nb_2_CT_x_ promotes the formation of oxygen vacancies in CeO_2_. The signal intensity of the CeO_2_/Nb_2_CT_x_ composite material at the g value was significantly higher than that of pure CeO_2_, conclusively proving that it has a higher O_V_ concentration [34]. This enhancement stems from the interaction between Nb_2_CT_x_ and CeO_2_, which promotes the formation of defects (oxygen vacancies) by altering the local electronic environment, thereby accelerating the charge transfer process. The increased O_V_ density can promote the adsorption of NH_3_ molecules more effectively, thereby enhancing the sensing response. The CeO_2_ and Nb_2_CT_x_ heterojunction optimizes the interfacial electron transport. The work function difference between CeO_2_ and Nb_2_CT_x_ is only 0.12 eV, which means that the Schottky barrier (SBH) formed at the interface is very small (Figure 2a_4_). Therefore, electrons can be transmitted more easily across interfaces, significantly enhancing the sensing capability. Nb_2_CT_x_ exhibits metallic properties (the valence band and conduction band overlap at the Fermi level) [35]. After the formation of the CeO_2_/Nb_2_CT_x_ heterojunction, the energy bands at the interface overlap, presenting metallic characteristics, which makes it easy for electrons to transfer from CeO_2_ to Nb_2_CT_x_, thereby achieving an excellent NH_3_ sensing response at room temperature.

Liu et al. [36] formed MXene/In_2_O_3_ heterostructures through a simple hybridization process, enabling In_2_O_3_ nanoparticles to be dispersed and encapsulated on the surface, as well as partially embedded in the intermediate layers of layered MXene. This layered structure disperses In_2_O_3_ nanoparticles between MXene sheets, effectively preventing layering while maintaining open diffusion channels and providing a certain degree of physical antioxidant protection. At room temperature, the response of the MXene/In_2_O_3_ hybrid sensor to 20 ppm ammonia significantly increased from 3.6% to 100.7%. A heterostructure is formed between MXene and In_2_O_3_. Since the Fermi energy level of N-type In_2_O_3_ is higher than that of P-type MXene (Ti_3_C_2_T_x_), electrons will transfer from In_2_O_3_ to MXene until the Fermi energy levels of the two materials reach equilibrium (Figure 2b_1_). This process will form a heterostructure at the interface between MXene and In_2_O_3_. This leads to an increase in the concentration of free electrons in MXene. More oxygen in the air can be pre-adsorbed on the surface of the sensing membrane and capture electrons to form ionized oxygen molecules (O_2_*^−^*) (Figure 2b_2_). Due to the increase in the amount of adsorbed O_2_*^−^*, the reaction intensifies. In situ infrared spectroscopy of MXene/In_2_O_3_ exposed to NH_3_ indicates that gaseous nitric oxide is produced during the sensing process. In addition, the response of MXene/In_2_O_3_-based sensors to ammonia gas increases with the increase in the relative humidity of the mixed gas. This can be explained as the “solvent”-assisted catalytic effect [37]. At room temperature, the generated NH^4+^ can actively interact with the O_2_*^−^* pre-adsorbed on the surface of MXene/In_2_O_3_ (Figure 2b_3_). Therefore, as humidity increases, it further promotes the gas-sensitive reaction, thereby releasing more electrons to the depletion region on the surface of MXene/In_2_O_3_, which improves the sensitivity. This sensor demonstrates the potential to detect NH_3_ in high-humidity atmospheres, such as for disease diagnosis in human respiration.

Tai et al. [38] fabricated a gas sensor based on Ti_3_C_2_T_x_ composite films loaded on TiO_2_ by a simple spraying method and studied its NH_3_ sensing performance at room temperature. The construction of TiO_2_/Ti_3_C_2_T_x_ composite films can enhance the NH_3_ gas sensitivity performance of Ti_3_C_2_T_x_ nanosheets (Figure 2c_1_). The emergence of this function was due to the dual role of titanium dioxide nanoparticles: (i) as physical spacers mitigating Ti_3_C_2_T_x_ restacking, and (ii) as interfacial passivation layers. Although excessive oxidation degrades performance, the controlled introduction of TiO_2_ at Ti_3_C_2_T_x_ interfaces or surfaces passivates reactive edges/sites. This inhibits subsequent bulk oxidation, ultimately enhancing the composite’s environmental stability. Researchers have conducted an in-depth analysis of the NH_3_ sensing response of the pure Ti_3_C_2_T_x_ gas sensor. The resistance of TiO_2_/Ti_3_C_2_T_x_ (≈650 Ω) is much smaller than that of pure TiO_2_ (≈21.3 kΩ), indicating that the electrical properties of TiO_2_/Ti_3_C_2_T_x_ depend mainly on Ti_3_C_2_T_x_. The gas response direction of the TiO_2_/Ti_3_C_2_T_x_ sensor is consistent with that of the Ti_3_C_2_T_x_ sensor. When N-type TiO_2_ comes into contact with Ti_3_C_2_T_x_, electrons on the TiO_2_ conduction band will migrate towards Ti_3_C_2_T_x_ due to its excellent metallic properties and high work function, thereby forming a self-built electric field (Schottky barrier) at its interface [39]. In the air, the hole density on the surface of Ti_3_C_2_T_x_ increases, while the electrons on the surface of TiO_2_ decrease. This is because the generation of adsorbed ionized oxygen consumes electrons, resulting in an increase in holes in Ti_3_C_2_T_x_, weakening the self-built electric field and reducing the resistance of TiO_2_/Ti_3_C_2_T_x_. Conversely, in NH_3_, due to the reduction in holes on the surface of Ti_3_C_2_T_x_ and the increase in electrons on the surface of TiO_2_, the self-built electric field will be enhanced, resulting in an increase in resistance (Figure 2c_2_) [40]. Therefore, the regulation of the self-built electric field (space charge layer) is thought to be the main reason for the enhanced NH_3_ sensing response of the TiO_2_/Ti_3_C_2_T_x_ gas sensor.

### 3.2. The Introduction of Two-Dimensional Materials

Integrating other 2D materials with MXenes, particularly TMDs such as MoS_2_, MoSe_2_, WS_2_, or ReS_2_, creates unique van der Waals heterostructures. The TMD nanosheets act as ideal spacers between MXene layers, efficiently suppressing restacking and maximizing the exposed surface area. Critically, TMDs exhibit superior oxidation stability compared to MXenes. When grown vertically or conformally on the surface of MXene, they form a protective layer that significantly slows down the oxidation of the lower MXene, expands the hierarchical diffusion channels, and shortens the response time.

**Figure 3:**
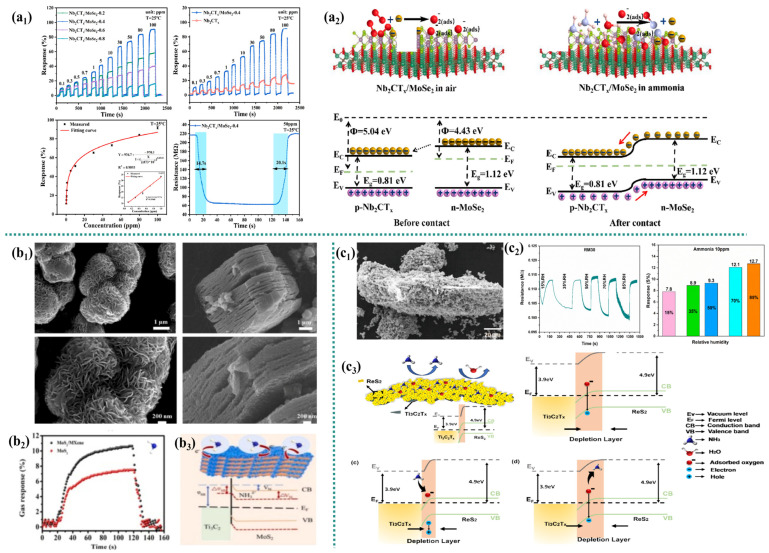
(**a**) Nb_2_CT_x_/MoSe_2_ composite [41]: (**a_1_**) gas sensitivity performance, and (**a_2_**) the band gap and work function for samples; (**b**) MoS_2_ nanosheets on MXene (Ti_3_C_2_) nanoribbons [43]: (**b_1_**) SEM, (**b_2_**) response–recovery curve, and (**b_3_**) band diagram; (**c**) ReS_2_/Ti_3_C_2_T_x_ heterostructures [47]: (**c_1_**) SEM, (**c_2_**) gas sensitivity performance, and (**c_3_**) band diagram.

Xiong et al. synthesized a Nb_2_CT_x_/MoSe_2_ sensor using a one-step hydrothermal method and applied it to detect NH_3_ [41]. Compared with pure Nb_2_CT_x_, the sensor based on Nb_2_CT_x_/MoSe_2_ composite material has a more stable baseline resistance. The response to 50 ppm NH_3_ is increased by 3.5 times, and the response/recovery time is shortened by 56.4 s/32.1 s. In addition, the sensor’s response to NH_3_ (1 ppm, 50 ppm, and 100 ppm) varies by less than 10% within 90 days, demonstrating excellent stability (Figure 3a_1_). This represents a significant improvement over the original Nb_2_CT_x_. The main reason for using MoSe_2_ is that it has a dual function: it can prevent the restacking of Nb_2_CT_x_ and also form a protective and anti-oxidative barrier. The sensing behavior of Nb_2_CT_x_ exhibits the characteristics of P-type semiconductors, while the sensing behavior of MoSe_2_ exhibits the characteristics of N-type semiconductors. When P-type Nb_2_CT_x_ and N-type MoSe_2_ combine, they form a p-n heterojunction, enhance electron transfer, and form an electron depletion layer. The work functions of Nb_2_CT_x_ and MoSe_2_ are 5.04 eV and 4.43 eV, respectively, and the band gaps (E_g_) are 0.81 eV and 1.12 eV, respectively [42]. When they come into contact, electrons transfer from the conduction band of MoSe_2_ to that of Nb_2_CT_x_. This electron transfer causes the energy band within the depletion layer to bend until equilibrium is reached at the Fermi level, forming a p-n heterojunction at the interface between the materials. The formation of heterojunctions enhances the electron transfer ability within Nb_2_CT_x_/MoSe_2_, facilitating the transfer of surface charges and thereby improving the gas-sensitive performance of the sensor. It is notable that the p-n heterojunctions formed on the surface of the composite material significantly increase the adsorption energy for NH_3_, thereby significantly improving the selectivity of the sensor for NH_3_ (Figure 3a_2_). On the other hand, the excellent ammonia-sensing performance of the Nb_2_CT_x_/MoSe_2_ sensor may also be attributed to the in situ growth of MoSe_2_ nanoflowers. In situ-grown MoSe_2_ nanoflowers on Nb_2_CT_x_ not only increase the specific surface area but also create a physical barrier that minimizes direct contact between Nb_2_CT_x_ nanosheets and the ambient environment. This thereby enhances resistance to interlayer restacking and oxidative degradation while providing additional ammonia adsorption sites, ultimately improving the ammonia sensing response of the composite material. To sum up, these factors jointly promote the enhancement of the NH_3_ sensing performance of Nb_2_CT_x_/MoSe_2_ sensors.

Guo et al. [43] grew MoS_2_ nanosheets in situ on MXene (Ti_3_C_2_) nanoribbons for ammonia (NH_3_) detection. The sensor exhibited a significant gas response to 100 ppm NH_3_ (~10%). The sensor maintained its performance at 1 ppm NH_3_, with a gas response of 2.5%, a response time of 10 s, and a recovery time of 7 s (Figure 3b_1_). The main gas-sensitive mechanism is thought to be surface charge transfer and a modulation in the Schottky potential barrier junction between MoS_2_ and Ti_3_C_2_ [44]. The Fermi level of Ti_3_C_2_ is located between the conduction and valence bands of MoS_2_. When Ti_3_C_2_ comes into contact with MoS_2_, a Schottky barrier (SB) junction is formed, causing the MoS_2_ energy band to bend. During the gas response process, as the electron concentration changes, the built-in potential (Vbi) and the width of the Schottky barrier adjust, and the Fermi level rises to a position far from the MoS_2_ valence band (Figure 3b_2_). The Schottky barrier height, Vbi, and the current formed at MoS_2_/Ti_3_C_2_ increase, significantly increasing the sensitivity of the gas sensor. The high response to NH_3_ might be due to the stronger electron-donating effect of NH_3_ [45]. Furthermore, hydrogen bonds are easily formed between Ti_3_C_2_ and NH_3_, achieving strong charge transfer and causing the SB junction [46] to shift. The synergistic effect between MoS_2_ and Ti_3_C_2_ enhances both gas sensitivity and stability. This improvement stems from MoS_2_’s dual function as a spacer, suppressing Ti_3_C_2_ restacking while providing an oxidation-resistant barrier due to its inherent chemical stability.

Gasso et al. [47] vertically grew ReS_2_ nanosheets on the surface of Ti_3_C_2_T_x_ sheets through hydrothermal synthesis, forming ReS_2_/Ti_3_C_2_T_x_ heterostructures. Compared with ReS_2_ and Ti_3_C_2_T_x_, the specific surface area of the heterostructure increased by 1.3 times and 8 times, respectively. This is a direct result of ReS_2_ preventing the re-accumulation of MXene, potentially enhancing the adsorption sites of active gases. The electrical study of the sensor based on ReS_2_/Ti_3_C_2_T_x_ shows that when the relative humidity at room temperature is between 15 and 85% and the NH_3_ concentration is 10 ppm, the selectivity of the ReS_2_/Ti_3_C_2_T_x_ sensor is enhanced, and the sensing response range is 7.8–12.4% (Figure 3c_1_). This enhancement is attributed to the unique heterogeneous interface and modified surface chemistry, which collectively mitigate humidity interference. Growing edge-exposed ReS_2_ nanosheets on Ti_3_C_2_T_x_ thin sheets with high electrical conductivity and a large surface area provides a large number of active sites for gas molecules and facilitates the transport of charge carriers during the sensing process, thereby improving the gas sensing characteristics. Additionally, the ReS_2_ layer serves as a protective layer, significantly helping to prevent the oxidative degradation of Ti_3_C_2_T_x_. The physical barrier effect was a key factor in achieving the stable performance of heterogeneous structures. The presence of negative charges on the ReS_2_/Ti_3_C_2_T_x_ heterostructure due to –O and –OH can further enhance the interaction with NH_3_ through hydrogen bonds and electron-donating mechanisms, making the material more sensitive to NH_3_. Therefore, the synergistic effect of ReS_2_ and Ti_3_C_2_T_x_ leads to the synthesis of stable ReS_2_/Ti_3_C_2_T_x_ heterostructures, promotes more interactions between the sensing layer and the target gas, and accelerates the adsorption and desorption processes of the gas (Figure 3c_3_).

### 3.3. The Introduction of Polymers

Polymers (including PANI, PEDOT:PSS, PPy, and PU) have been utilized to construct 3D networks through techniques such as electrospinning and in situ polymerization, physically separating MXene nanosheets and effectively preventing re-accumulation while maintaining porosity and optimizing gas diffusion pathways. Crucially, polymeric encapsulation forms conformal coatings on MXene sheets, thereby establishing a physical barrier against oxygen and water vapor ingress. This significantly enhances oxidation resistance and long-term stability. Furthermore, the inherent hydrophobicity of polymers such as polyurethane (PU) and polypyrrole (PPy) effectively repels water molecules, mitigating humidity interference. Concurrently, polymers introduce the essential mechanical flexibility for wearable sensor applications; sensors made of these composite materials can withstand higher tensile strains and repeated bending cycles while maintaining stable response values.

**Figure 4:**
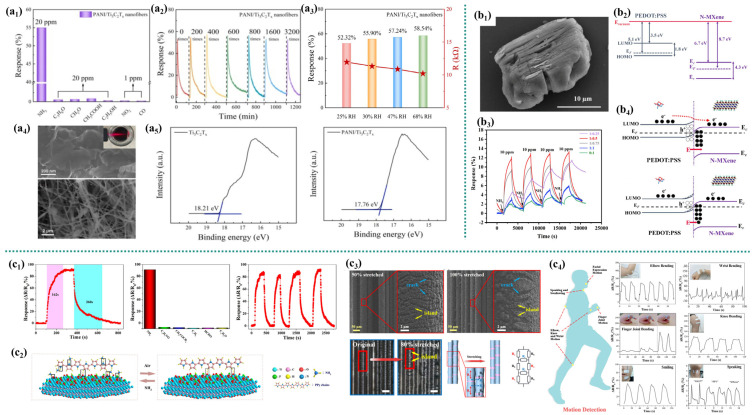
(**a**) Polyaniline/Ti_3_C_2_T_x_ composite nanofiber [47]: (**a_1_**) the selectivity for samples, (**a_2_**) mechanical flexibility, (**a_3_**) the response for samples under different humidity, (**a_4_**) SEM, (**a_5_**) and the UPS for samples; (**b**) MXene to PEDOT: PSS components [56]: (**b_1_**) SEM, (**b_2_**) schematic diagram, (**b_3_**) the response–recovery curves under different humidity conditions, and (**b_4_**) heterojunction band bending diagram; (**c**) PU-supported Ti_3_C_2_T_x_/TiO_2_/PPy [59]: (**c_1_**) the gas-sensing performance of the gas sensor, (**c_2_**) ammonia adsorption on the material surface, (**c_3_**) SEM, and (**c_4_**) monitoring of human body shape changes.

Yang et al. [48] combined polyaniline (PANI) with Ti_3_C_2_T_x_ nanosheets through electrospinning to construct a flexible reticular polyaniline/Ti_3_C_2_T_x_ composite nanofiber sensor (Figure 4a_4_). This new type of flexible PANI/Ti_3_C_2_T_x_ sensor has a higher NH_3_ sensing response at 25 °C (2.3 times higher at 20 ppm) (Figure 4a_1_). Under various bending angles (maximum compression: 150°) and different bending times (maximum bending: 3200 times), the PANI/Ti_3_C_2_T_x_ flexible sensor maintains a stable sensing behavior at 20 ppm NH_3_, demonstrating its excellent flexible bending stability (Figure 4a_2_). As the relative humidity increases, the sensor’s response to 20 ppm NH_3_ slightly increases. H_2_O may affect the degree of polyaniline protonation. Under high RH conditions, when H_2_O molecules are absorbed by the PANI chain, the number of conductive ions increases and the sensor resistance decreases [44,49] (Figure 4a_3_). Polyaniline’s protonation/deprotonation processes are used to explain its detection mechanism [50,51]. Polyaniline is a conductive P-type semiconductor that exists in the form of emerald imine salts. Polyaniline nanofibers generate N+ –H bonds (protonation) during the oxidative polymerization process. When NH_3_ is introduced, deprotonation occurs, and the electrical conductivity changes due to the change from the conductive emerald salt form to the insulating emerald base form [51,52]. Specifically, NH_3_ molecules react with protons (H^+^) of = NH^+^− and –NH_2_^+^− from PANI to form NH_4_^+^. Therefore, as the NH_3_ concentration increases, the number of holes in the PANI sensor decrease and the resistance increases [53,54]. When the sensor is placed in the air, the reaction process reverses, resulting in a decrease in the sensor’s resistance. The enhanced sensing response of the PANI/Ti_3_C_2_T_x_ sensor can be attributed to the synergistic effect of PANI and Ti_3_C_2_T_x_ nanosheets in the composite nanofibers. The Schottky junction formed at the interface between Ti_3_C_2_T_x_ and PANI enhances the resistance modulation capability of the flexible sensor [48]. The work functions of Ti_3_C_2_T_x_ and PANI/Ti_3_C_2_T_x_ composite nanofibers are 2.99 eV and 3.44 eV, respectively (Figure 4a_5_). Therefore, the holes in PANI H^+^ and the electrons in Ti_3_C_2_T_x_ move in opposite directions until the new Fermi level reaches equilibrium. The adsorption of NH_3_ by the pore accumulation layer in the interface area is greater, as is the probability of the induction reaction with NH_3_ molecules [55]. This leads to a further reduction in the conductive channels and an additional increase in resistance, corresponding to higher NH_3_ sensing responses in PANI/Ti_3_C_2_T_x_ nanofibers. Secondly, the increase in the protonation degree of polyaniline in PANI/Ti_3_C_2_T_x_ composite nanofibers is an important reason for the enhanced NH_3_ response. The total relative ratios of =NH_2_^+^− and –NH_2_^+^− in polyaniline powder of polyaniline nanofibers increased from 34.16% to 53.68%, indicating that the protonation degree of polyaniline was enhanced due to the construction of the fiber structure. The increase in protonation degree significantly enhances the response to NH_3_. The interconnected network structure of PANI/Ti_3_C_2_T_x_ nanofibers offers increased adsorption sites for NH_3_ molecules, thereby enhancing the NH_3_ sensing response.

Qiu et al. [56] combined the organic conductive conjugated polymer PEDOT:PSS with inorganic nitrogen-doped transition metal carbides and nitrides to conduct chemical NH_3_ resistance sensing at room temperature. The NH_3_ sensing performance of the composite membrane sensor is superior to that of a single N-MXene (Figure 4b_3_). It has a higher response speed and a faster response/recovery speed, as well as good repeatability, stability, and selectivity. The added N atoms are excellent electron donors, promoting the electron transfer reaction and increasing the adsorption sites. The corresponding internal field modulation in the heterojunction is the main reason for reversible NH_3_ sensing (Figure 4b_4_). PEDOT: The highest occupied molecular orbital (LUMO) and the lowest occupied molecular orbital (LUMO) of PSS are located at −5.3 and −3.5 eV, respectively, and the work function is 5.1 eV [57,58] (Figure 4b_2_). The appropriate incorporation of N-MXene promotes the kinetic equilibrium. On the one hand, the incorporated N atoms are excellent electron donors, capable of activating electron transfer reactions and increasing the number of adsorption sites. On the other hand, the generated TiO_2_ nanoparticles widened the interlayer spacing and may have inhibited the restacking of MXene during the film deposition process (Figure 4b_1_), thereby achieving relatively free gas diffusion/penetration within the sensing layer, followed by rapid reaction kinetics. Furthermore, these two aspects jointly promote the adsorption of NH_3_, thereby enhancing the response. In addition, N-MXene occupies some high-energy adsorption sites in the PEDOT: PSS matrix, which can also accelerate the response and recovery speed.

Zhao et al. [59] proposed a multifunctional flexible sensor based on PU-supported Ti_3_C_2_T_x_/TiO_2_/PPy yarn for ammonia (NH_3_) gas sensing and human motion detection (Figure 4c_1_). Adding polypyrrole (PPy) to the Ti_3_C_2_T_x_ MXene structure and optimizing the content of titanium dioxide (TiO_2_) through different hydrothermal times significantly improved the gas sensitivity performance of Ti_3_C_2_T_x_ MXene. This sensor demonstrates excellent sensitivity and selectivity for NH_3_, featuring a rapid response and recovery time. The gas-sensitive response of Ti_3_C_2_T_x_/TiO_2_/PPy nanocomposites to NH_3_ may be due to the adsorption/desorption of NH_3_ on the surface of the nanocomposites, thereby leading to the deprotonation/protonation process. The high electron mobility of Ti_3_C_2_T_x_ facilitates the rapid transport of carriers in nanocomposites, thereby achieving better sensing behavior. Secondly, the two-dimensional material Ti_3_C_2_T_x_ has a large specific surface area, providing sufficient adsorption sites for ammonia molecules on the surface of nanocomposites. Furthermore, the TiO_2_ in Ti_3_C_2_T_x_/TiO_2_/PPy expands the interlayer spacing of Ti_3_C_2_T_x_, providing more active sites for the adsorption of NH_3_. The oxygen absorbed from the air carried away the electrons of TiO_2_, and its holes were transferred to Ti_3_C_2_T_x_. Furthermore, the high gas sensitivity response may be related to the synergistic effect between Ti_3_C_2_T_x_ and PPy. The formation of hydrogen bonds between Ti_3_C_2_T_x_ and PPy may be related to the –H group on the PPy chain and the –OH group on the surface of Ti_3_C_2_T_x_ [60] (Figure 4c_2_). When Ti_3_C_2_T_x_/TiO_2_/PPy nanocomposites are exposed to an NH_3_ atmosphere, NH_3_ can form hydrogen bonds with bare PPy, Ti_3_C_2_T_x,_ and TiO_2_. In addition, the original hydrogen bonds among Ti_3_C_2_T_x_, TiO_2,_ and PPy will be broken, resulting in more NH_3_ molecules adsorbing onto the surface of the composite material, further increasing the resistance.

This flexible sensor exhibits excellent sensitivity and stability, primarily due to the Ti_3_C_2_T_x_/TiO_2_/PPy network structure on the PU yarn surface (Figure 4c_3_). Initially, in a pre-stretched state, the yarn surface displays numerous closed cracks and fewer open cracks. During stretching, new cracks emerge on the composite conductive layer. As strain increases, existing cracks widen and new ones form as the PU yarn elongates, causing the conductive components to separate. This outstanding performance provides a technical foundation for monitoring fundamental human physiological activities. Assembled Ti_3_C_2_T_x_/TiO_2_/PPy composites on various body parts effectively detect motion and physiological signals. During wearer movement, the material stretches, increasing the sensor’s ΔR/R_0_, which enables the monitoring of motions from subtle to significant, such as finger joint bending from slight angles to 90°. Its high strain sensitivity allows effective monitoring of body deformations, indicating broad application prospects in intelligent IoT devices and human–computer interactions. Furthermore, the Ti_3_C_2_T_x_/TiO_2_/PPy nanocomposite sensitively detects subtle movements like smiling and speaking. When subjects uttered polysyllabic words (e.g., “HAUT”, “PPY”, “MXene”), the sensor provided distinct signal feedback (Figure 4c_4_). This successful vocal cord motion detection expands its potential for speech recognition applications.

### 3.4. The Introduction of Precious Metals

The introduction of precious metal nanoparticles (NPs) such as Ag, Au, Pt, Ru, and Pd offers unique advantages. While primarily known for catalytic enhancement, these NPs can also act as nanoscale spacers, dispersed on the MXene surface or within layers, modestly contributing to mitigating restacking. More significantly, certain noble metals (e.g., Pt, Au, Ru) possess high chemical stability and can act as oxidation catalysts or sacrificial sites. They may scavenge reactive oxygen species or alter surface reaction pathways, thereby protecting MXene from oxidation. Furthermore, their catalytic activity can be harnessed to decompose interfering molecules such as water vapor or promote specific reactions that lessen humidity impact (e.g., Ru facilitating water dissociation). They also form Schottky junctions that amplify signals and enhance the sensitivity of sensors.

**Figure 5:**
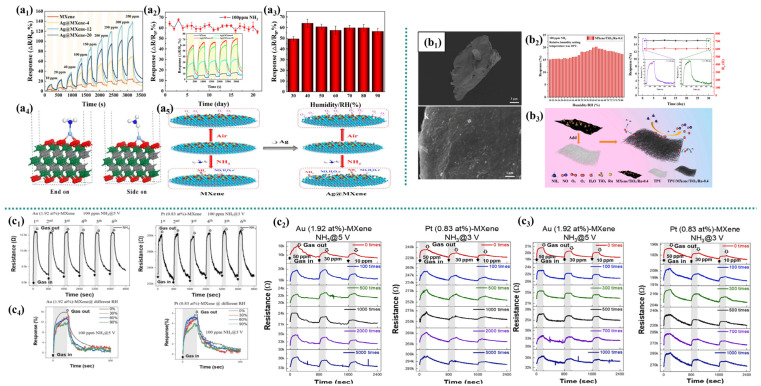
(**a**) Ag@MXene [61]: (**a_1_**) schematic diagram of multiple response recovery curves, (**a_2_**) 20-day response line chart, (**a_3_**) response graphs at different humidity levels, (**a_4_**) DFT calculated adsorption diagram, and (**a_5_**) Ag sensitization mechanism; (**b**) MXene/TiO_2_/Ru [63]: (**b_1_**) SEM, (**b_2_**) the response graphs of the sensor under different humidity conditions and the 30-day response graph, and (**b_3_**) schematic diagram of the sensor and the gas-sensitive performance; (**c**) Au/MXene or Pt/MXene [42]: (**c_1_**) long-term cycle diagram, (**c_2_**) response graphs of different bending cycles, (**c_3_**) response graphs of different bending cycles, and (**c_4_**) the response graphs of the sensor under different humidity conditions.

Zhao et al. [61] directly prepared Ag nanoparticles (NPs) loaded with Ti_3_C_2_T_x_ MXene (Ag@MXene) through the auto redox reaction of Ti_3_C_2_T_x_ with Ag^+^. The Ag@MXene sensor exhibits a 7-fold higher response to NH_3_ at room temperature compared to pure MXene (Figure 5a_1_). This enhancement primarily stems from Ag NPs inserted between Ti_3_C_2_T_x_ layers via autoredox reaction, acting as nanospacers that inhibit MXene restacking and thereby increasing specific surface area and active sites for NH_3_ adsorption. The composite maintained stable performance over a 21-day test (Figure 5a_2_). DFT calculations confirm stronger NH_3_ adsorption on Ag@MXene (−1.501 eV) versus pure MXene (−0.369 eV), indicating that Ag protects MXene active sites (Figure 5a_4_). Furthermore, Ag NPs catalyze the dissociation of O_2_ into reactive O_2_^−^ [62] (Figure 5a_5_), promoting NH_3_ oxidation and reducing consumption of low-valent surface Ti (Ti^2+^/Ti^3+^). Across a 30–90% RH range, the response of Ag@MXene to 100 ppm NH_3_ consistently exceeds 49.4% (Figure 5a3), surpassing the reported responses of MXene-based sensors. Although the response fluctuates at high humidity, the minimum value remains superior. Ag’s hydrophobicity partially counteracts MXene’s hydrophilic groups (–OH, –F), reducing H_2_O competitive adsorption. Additionally, the catalytic effect favors the reaction of NH_3_ with O_2_^−^, thereby mitigating humidity interference.

Zhao et al. [63] utilized TPU fiber pads as the base material and synthesized MXene/TiO2/Ru composites through a simple and effective method, subsequently loading them onto the fiber pads. The TPU/MXene/TiO_2_/Ru flexible sensor features a wide tensile range, good durability, high sensitivity, and good air permeability. At a stretching speed of 0.5 mm/s, when the TPU/MXene/TiO_2_/Ru flexible sensor is stretched to 120% of its original length, its response can reach 80.4 k. The maximum response of this sensor to 100 ppm NH_3_ at room temperature is 15.06%, and it also has high selectivity and stability. Ru doping can reduce the activation energy required for the combination of NH_3_ with the TiO_2_/MXene surface, thereby enhancing the chemical adsorption of NH_3_. Furthermore, the catalytic activity of Ru as a catalyst promoted the decomposition of NH_3_, significantly reducing the surface reaction barrier between the composite material and NH_3_, thereby enhancing the surface reaction activity [64]. Ru nanoparticles are uniformly dispersed between MXene/TiO_2_ layers, forming a stable composite structure that effectively inhibits the stacking of MXene sheets (Figure 5b_1_). This increases the active sites of the composite material for the target gas molecules and enhances the sensing ability of the composite material. The attenuation in the sensor’s response to 100 ppm NH_3_ within 30 days was extremely small, demonstrating that Ru enhanced the material stability. At a relative humidity of 30% to 62% (RH), the sensor’s response to NH_3_ increases. This is because the hydrophilicity of MXene promotes the combination of H_2_O and NH_3_. When RH > 62%, the decline in the response of Ru composites is much lower than that of pure MXene (Figure 5b_2_). This is attributed to Ru regulating the hydrophilic/hydrophobic balance on the material surface and catalyzing the synergistic reaction between water molecules and NH_3_, thereby alleviating the competitive adsorption of water molecules on active sites in high humidity (Figure 5b_3_).

Nam et al. [42] prepared separated MXene nanosheets after etching and successfully decorated them with Au or Pt nanoparticles. The sensor’s response value to ammonia gas reached 16%@100 ppm NH_3_ (Au-modified nanosheet), which is superior to pure MXene (6.13%@500 ppm). MXene maintains an accordion-like layered structure, providing open gas channels and increasing the specific surface area and active sites to enhance the diffusion efficiency of NH_3_. Au/Pt NPs were chemically reduced onto the surface of Ti_3_C_2_T MXene to form nanoscale spacers, effectively inhibiting the stacking of MXene sheets [65]. The sensor maintains stable performance without structural degradation after long-term cycling (Figure 5c_1_) and 1000 bends/tilts (Figure 5c_2_–c_3_). This is because Au/Pt NPs enhance conductivity [66], enabling the sensor to heat up through Joule heat at a low voltage (3–5 V) and avoiding high-temperature oxidation. Pt catalyzes the dissociation of NH_3_ [67,68], reducing direct oxidation on the surface of MXene. Under a high-humidity environment of 90% RH, the sensor still maintains a detectable response to 100 ppm NH_3_ (Figure 5c_4_), outperforming most MXene-based sensors. The hydrophobicity of Au/Pt NPs partially shields the hydrophilic groups (–OH, –F) of MXene, reducing the competitive adsorption of water molecules. Pt preferentially catalyzes the reaction between NH_3_ and O_2_^−^ in a humid environment, suppressing the interference of water molecules. In addition, self-heating (Joule heat (3–5 V)) promotes the desorption of water molecules and maintains the sensing activity.

To better understand the room-temperature ammonia sensing performance of different MXene-based composites, Table 1 summarizes the research progress reported in the literature.

## 4. Applications of MXene-Based Ammonia Gas Sensors

Due to its unique structure, MXene offers significant advantages for gas detection. Its single-layer or few-layer stacked structure endows this material with an extremely high specific surface area, thereby creating an extremely rich number of active sites for the adsorption of gas molecules. More importantly, during the preparation of MXene, by selectively etching various termination groups (such as –O, –OH, and –F) introduced on its surface, not only are the electronic properties of the material regulated, but active sites for ammonia molecule adsorption and interaction are also introduced, laying the foundation for high sensitivity and potential selectivity. In terms of electrical properties, MXene (especially Ti_3_C_2_T_x_) exhibits high electrical conductivity approaching that of metals, making it easy to capture the electrical signal changes in gas detection. This outstanding characteristic is crucial for constructing highly sensitive resistance/conductive ammonia gas sensors. At the same time, its high conductivity makes it possible to operate at room temperature, which is a revolutionary advantage. It completely eliminates the need for high-temperature heating elements, which are relied upon by traditional semiconductor metal oxide ammonia gas sensors, significantly reducing energy consumption, improving safety, and enabling it to adapt to extreme or hazardous environments, such as low-temperature cold chains, mines, and chemical production sites. Additionally, the MXene material also exhibits excellent mechanical flexibility and strength, allowing for gas detection applications in wearable fields. Its films or composite materials can withstand repeated bending, folding, and even a certain degree of stretching deformation without losing their functional integrity. These sensors can be directly attached to complex, curved surfaces (such as industrial pipes and equipment shells) or human surfaces (such as skin and clothing), greatly expanding the deployment range of sensor nodes and the diversity of monitoring modes.

The synergistic effects of the above-mentioned structure and its electrical and mechanical properties jointly lead to outstanding advantages and broad application prospects of MXene-based ammonia gas sensors in multiple application fields. Its high sensitivity, room-temperature operation capability, flexible compatibility, and adaptability to harsh environments make it demonstrate great potential to replace or innovate traditional detection technologies in many critical scenarios where efficient, safe, and convenient ammonia monitoring is urgently needed. This section will systematically elaborate on the research progress, performance, and practical value of ammonia sensors based on MXene in the following core application scenarios, encompassing dimensions such as industrial production and safety protection, food safety assurance, health management in agriculture and animal husbandry, and wearable health monitoring in medical diagnostics.

### 4.1. Industrial Production and Safety Protection

Ammonia is an important raw chemical material and refrigerant. In the production processes of ammonia, urea, nitric acid, and fertilizers, as well as in large industrial refrigeration systems that use ammonia as a refrigerant (such as petrochemical plants and cold storage facilities), it is crucial to accurately monitor potential ammonia leakage in real time. The high sensitivity (capable of detecting ammonia as low as the ppb level) and rapid response/recovery characteristics of MXene sensors make them an ideal choice for early leakage warning, effectively preventing fires, explosions, and personnel poisoning accidents. In industrial environments involving the production, storage, transportation, or use of ammonia, it is a mandatory safety requirement to equip workers with portable or fixed ammonia monitors. MXene-based sensors, due to their performance advantages and potential flexibility and low power consumption characteristics, are suitable for development into wearable personal exposure monitoring devices.

**Figure 6:**
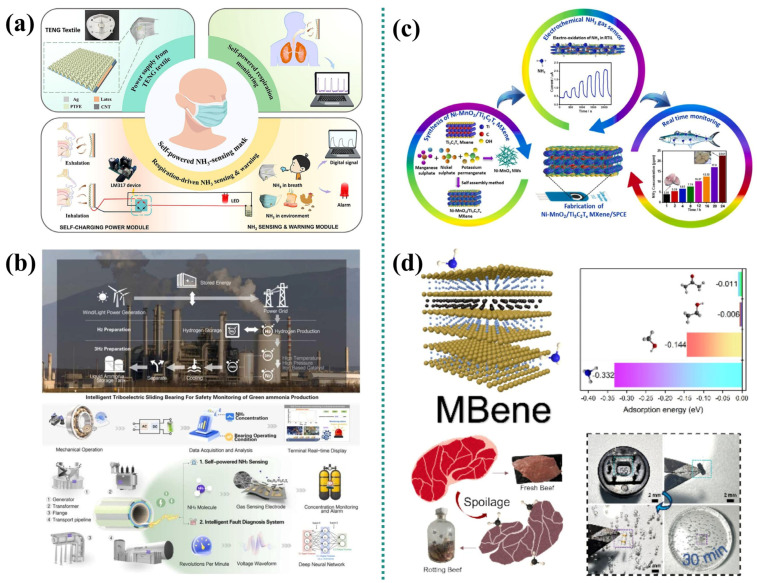
(**a**) The self-driven data transmission unit of the high-performance Ga_2_O_3_/MXene ammonia sensor automatically regulates and stores the output schematic diagram of the independent rotating bucket TENG [76]; (**b**) schematic diagram of the sensing and early warning principle of the external environment NH_3_ for the smart mask based on the Ti_3_C_2_T_x_/PANI sensor [77]; (**c**) schematic diagram of real-time monitoring of the freshness of packaged food using Ni-MnO_2_/Ti_3_C_2_T_x_ ammonia gas sensor [78]; and (**d**) schematic diagram of the gas released during the spoilage process of beef detected by MBene gas sensor [79].

Wang et al. [76] developed a high-performance ammonia sensor based on Ga_2_O_3_/MXene nanocomposites (Figure 6a). They created a self-driven data transmission unit, which autonomously regulates and stores the independent rotating bucket TENG output for sensing and monitoring mechanical operation, and proposed a neural network algorithm for predicting mechanical bearing faults. Combined with field data, the accuracy rate is 99%. This integrated system provides a practical and feasible solution for sensing gasses and diagnosing mechanical bearing faults during green ammonia synthesis production, thereby achieving the sustainable and safe development of green ammonia synthesis production. Huang et al. [77] established a self-powered NH_3_ sensing system with continuous power supply capability. A fully self-powered smart mask was developed by integrating TENG textiles and NH_3_ sensing modules, achieved by placing Ti_3_C_2_T_x_/PANI onto the mask. Due to the continuous energy supply of the TENG fabric during human breathing, combined with the excellent sensing performance of the NH_3_ sensing module, the smart mask possesses precise sensing and early warning capabilities for both human breathing and NH_3_ in the external environment, eliminating the need for a bulky external power source. This wearable NH_3_ sensing device, which is based on green energy and exhibits both good sensing performance and ecological friendliness, has great application potential in aspects such as early warning of excessive NH_3_ in the external environment (Figure 6b).

### 4.2. Food Safety Monitoring

During the proteolytic degradation of protein-rich foods (e.g., meat, poultry, fish, seafood, and dairy products), microbial activity facilitates the decomposition of proteins into various amine compounds. Ammonia constitutes a critical volatile marker and metabolic byproduct indicative of this spoilage process. Trace amounts of gaseous ammonia are liberated during the initial phases of spoilage, with levels exhibiting a significant positive correlation with an increased spoilage severity. Conventional detection methodologies, including sensory evaluation and laboratory assays, are constrained by inherent limitations such as temporal lag, subjective interpretation, and operational complexity. MXene-based ammonia gas sensors address these limitations through their exceptional sensitivity (detection limits extending to ppb levels), enabling the identification of trace ammonia emissions during incipient spoilage stages, significantly preceding detection via human olfaction or visual inspection. Consequently, these sensors facilitate early-stage spoilage warning systems. Implementation of this technology is imperative for enhancing food safety protocols, mitigating risks associated with foodborne pathogens, and reducing food waste throughout the supply chain.

Mari et al. [78] synthesized Ni-MnO_2_/Ti_3_C_2_T_x_ MXene nanocomposites for ammonia detection. Given that NH_3_ is a key indicator of food spoilage, such gas sensors enable early-stage spoilage detection, thereby enhancing food safety and quality. The researchers demonstrated the real-time monitoring of packaged food freshness utilizing the Ni-MnO_2_/Ti_3_C_2_T_x_ MXene sensor. Its capability to detect NH_3_ indicates significant potential as a valuable tool for ensuring food safety in practical applications (Figure 6c). Separately, Yao et al. [79] reported a green, fluorine-free synthesis process for layered transition metal boride MoAl_1-x_B (MBene) nanomaterials. Combined experimental and theoretical studies revealed that the MBene gas sensor exhibits unique selectivity towards NH_3_ at room operating temperatures. Owing to its abundant active sites and vacancy defects, the sensor achieves a response of 10.9% to 50 ppm NH_3_. This performance enables the detection of gases released during beef spoilage processes (Figure 6d).

### 4.3. Precise Management of Agriculture and Animal Husbandry

Excessive application of nitrogen fertilizer in agricultural systems can lead to the large-scale volatilization of soil ammonium nitrogen (NH_4_^+^) in the form of gaseous ammonia. This process constitutes the main source of global anthropogenic ammonia emissions, accounting for more than 80% of the total burden. The result of this volatilization is a significant loss of fertilizer efficiency, manifested as an unsatisfactory nitrogen use efficiency (NUE), usually only 30% to 50%. This not only represents considerable economic waste but also exacerbates secondary environmental impacts, particularly through the acceleration of atmospheric fine particulate matter (PM 2.5) formation and the process of environmental acidification. In large-scale livestock and poultry farms (such as poultry houses and pigsties), animal excrement is decomposed by microorganisms to produce a large amount of ammonia. Long-term exposure to a high concentration of ammonia gas environment (>25 ppm) can induce respiratory diseases in animals, reduce feed conversion rate, suppress immune function, and ultimately lead to a decline in production performance (such as a decrease in the egg production rate of laying hens and a reduction in daily weight gain of pigs).

To address this critical issue, flexible ammonia sensors based on MXene can be strategically integrated into soil probes or distributed on-site Internet of Things (IoT) nodes. This integration can continuously and with high resolution quantify the ammonia volatilization fluxes at the soil-atmosphere interface after fertilization events (expressed as μg NH_3_ m^−2^h^−1^). MXene-based ammonia gas sensor networks should be deployed in key areas within the shed (ventilation dead corners, manure and sewage areas, and animal activity layers) to monitor the spatial distribution and dynamic changes in ammonia gas in real time.

**Figure 7:**
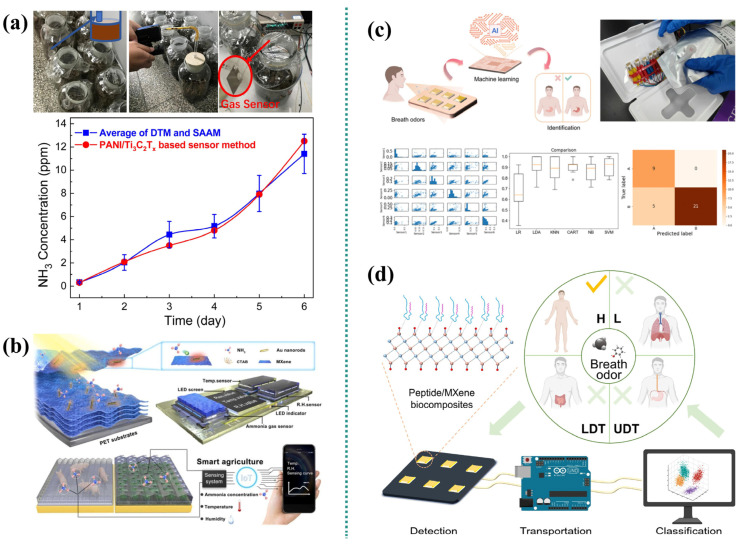
(**a**) Schematic diagram of an agricultural simulation experiment of a flexible chemical gas-resistant sensor based on polyaniline (PANI)/Ti_3_C_2_T_x_ hybrid sensitive film [48]; (**b**) schematic diagram of the application of plasma-functionalized MXenes light-driven ammonia sensors in intelligent agriculture [80]; (**c**) schematic diagram of the bionic sensor array based on Pd-Au/MXene sensor for distinguishing breath samples from healthy individuals and gastric cancer patients [81]; and (**d**) schematic diagram of a non-invasive early tumor diagnosis model based on polypeptide MXene biosensors [82].

Li et al. [48] developed a flexible chemical resistance gas sensor based on a polyaniline (PANI)/Ti_3_C_2_T_x_ hybrid sensitive film for monitoring ammonia volatilization in agriculture (Figure 7a). This sensor features high sensitivity, low detection limit, good repeatability, high selectivity, and good air stability. It also exhibits excellent ammonia sensing performance in 20–80% relative humidity (RH) and within the temperature range of 10–40 °C. The feasibility of applying this sensor for monitoring ammonia volatilization was verified through agricultural simulation experiments. Zhou et al. [80] developed a light-driven ammonia sensor utilizing plasma-functionalized MXenes (metal carbides/nitrides) to enable ultra-sensitive, room-temperature NH_3_ detection for smart agriculture applications. The u/HT-Nb_2_CT_x_-based gas sensor exhibits trace NH_3_ detection capability with a low detection limit (LOD = 500 ppb) and full recovery characteristics. Its sensitivity to 100 ppm NH_3_ (demonstrated by an 80% response magnitude) is more than double that of comparable sensors lacking gold nanoparticle (Au NP) functionalization. The researchers further implemented a portable optical-drive NH_3_ monitoring alarm system. This integrated platform wirelessly combines the NH_3_ sensor with temperature and humidity sensors, connecting to a mobile interface to enable continuous 24-h environmental surveillance. System functionality was successfully validated through real-time monitoring of NH_3_ concentration, temperature, and humidity within operational pig housing and vegetable greenhouse environments. This demonstration confirms the system’s capability to address the escalating demands for precision environmental management in smart agriculture and enhanced environmental safety protocols (Figure 7b).

### 4.4. Medical Diagnosis and Health Monitoring

Human exhaled breath contains trace concentrations of ammonia (NH_3_), with dynamic variations in the concentration exhibiting correlation with specific pathophysiological states. For instance, elevated blood urea nitrogen (BUN) levels in patients with renal failure drive a significant increase in exhaled ammonia concentration. Similarly, Helicobacter pylori infection has been associated with detectable alterations in exhaled ammonia profiles. The exceptional sensitivity intrinsic to MXene-based gas sensors renders them highly suitable for development into portable, low-cost, non-invasive exhaled breath analysis platforms. Such devices hold significant promise for the early screening and auxiliary diagnosis of systemic conditions, including renal dysfunction, hepatic disorders, and gastrointestinal infections.

Furthermore, certain bacterial pathogens implicated in wound infections generate ammonia as a metabolic byproduct during proliferation. The integration of flexible MXene sensors within advanced wound dressings presents a theoretically feasible strategy for the real-time, in situ monitoring of trace ammonia emissions originating from the wound microenvironment. This approach exploits ammonia as an early-stage biochemical marker of incipient infection, thereby enabling timely clinical intervention and targeted therapeutic management.

Chen et al. [81] fabricated a Pd-Au/MXene sensor exhibiting enhanced gas-sensing performance via an in situ growth strategy. A bionic sensor array based on this material was developed and integrated into a real-time in situ sensing platform (IISP). Machine learning (ML) algorithms were further employed to augment the gas recognition capability of the IISP in complex environments. Owing to the electronic sensitization and catalytic effects of the noble metal sites, the Pd-Au/MXene nanocomposites demonstrated superior gas-sensing properties, achieving a response speed 2.73 times faster than pristine Ti_3_C_2_T_x_ MXene. Additionally, utilizing pattern recognition algorithms, this sensor array successfully discriminated against 14 common volatile organic compounds (VOCs) encountered in daily life. Ultimately, aided by ML, the IISP achieved an accuracy of 92.0% in distinguishing exhaled breath samples from healthy individuals and gastric cancer patients (Figure 7c). Separately, Hu et al. [82] developed a peptide-functionalized MXene biosensor with significant gas-sensing enhancements using a self-assembly approach. Leveraging these biosensors, a mimetic biosensor array (MBA) was constructed and integrated into a real-time test platform (RTP). Machine learning algorithms were also introduced to the RTP to improve its detection and recognition performance for exhaled volatile biomarkers. The synthesized peptide MXene biosensor exhibits specific binding affinity for target gas molecules, yielding a response 150% higher than that of unmodified MXene. This approach facilitated the development of a cost-effective and accurate model for non-invasive early tumor diagnosis (Figure 7d).

## 5. Current Challenges and Future Perspectives

Despite the significant progress and promising applications highlighted in the above sections, the development and widespread deployment of MXene-based room-temperature NH_3_ sensors still face several critical challenges. Addressing these challenges is essential to transition this technology from promising laboratory demonstrations to reliable, commercially viable devices. This section outlines the key remaining hurdles and proposes potential future research directions.

### 5.1. Persistent Challenges

The inherent susceptibility of MXenes, especially Ti_3_C_2_T_x_, to oxidation in ambient air and under operational conditions remains the most significant threat to long-term stability and sensor lifetime. Oxidation progressively converts the conductive MXene core into insulating TiO_2_, degrading conductivity and surface reactivity. While composite strategies (e.g., with TMDs, oxides, polymers) offer protection, achieving complete suppression of oxidation over years of operation, particularly under fluctuating temperature/humidity or in reactive gas environments, is unproven. The degradation kinetics and failure modes under real-world sensor operating stresses need deeper understanding. The scalable synthesis of inherently more stable MXenes (beyond Ti-based) or those with optimized terminations remains limited.

High and variable ambient humidity severely compromises NH_3_ sensing performance (sensitivity, selectivity, baseline stability) due to competitive adsorption of water molecules on MXene surfaces and within composites. Existing strategies (hydrophobic polymers, catalytic metals, heterojunction engineering) show improvement but often fall short in achieving negligible humidity dependence across a wide RH range (20–95%). Developing materials or sensor designs that are intrinsically resistant to humidity, or incorporating effective in situ humidity compensation methods without complex circuitry, is challenging.

Achieving high selectivity for NH_3_ against common interferents (e.g., ethanol, acetone, H_2_, CO, NO_2_, H_2_S, VOCs) present in target applications (breath, industrial exhaust, farms, and spoiled food) is difficult at room temperature due to similar adsorption energies or reaction pathways. Most studies report selectivity against a limited set of gases under controlled lab conditions. Performance validation in real, complex gas mixtures mimicking actual application scenarios is scarce. The fundamental mechanisms governing selectivity in MXene composites, particularly under humid conditions, require further investigation.

### 5.2. Promising Future Research Directions

Advanced materials should be designed to enhance the stability of sensors. We need to strengthen research on non-titanium-based MXenes (such as Mo_2_CT_x_, V_2_CT_x_, and Nb_2_CT_x_) or titanium-based MXenes with modified surface terminations (–Cl and –S), which are known to potentially have higher antioxidant properties. We also need to develop complex structures, such as encapsulating MXene cores in robust, impermeable shells (for example, formed by atomic layer deposition (ALD) of Al_2_O_3_, graphene, stable oxides such as Al_2_O_3_/ZrO_2_ or highly cross-linked polymers), which aim to provide the maximum environmental barrier protection without sacrificing gas permeability. Additionally, we should explore strategies to induce the formation of thin, natural, and stable passivation layers (for example, through controlled surface oxidation to form a protective TiO_2_ film) to prevent further overall degradation.

Composite membranes or coatings with specific pore diameters (for example, using metal–organic framework materials, carbon framework materials, or precisely sized polymers) should be designed to allow ammonia gas to diffuse while physically blocking larger water molecules. We should also develop sensor arrays combined with MXene composite materials, which exhibit different selectivities, and utilize powerful machine learning algorithms (such as deep learning) to analyze the complex response patterns (intensity, kinetics) in the arrays, thereby enabling reliable identification and quantification of ammonia gas in the presence of humidity and various interfering substances. The humidity sensors integrated on the chip can provide data for real-time compensation of the algorithm.

## 6. Conclusions

MXene-based sensors, due to their unique layered architecture, tunable surface chemistry, and exceptional electrical conductivity, have emerged as a frontline system for high-sensitivity ammonia detection. This study systematically examines the design strategies of MXene and its composites, as well as the mechanisms of MXene in sensing NH3 gas and recent advancements in its applications. Through structural engineering, heterojunction construction, and modulation of surface functional groups and active sites, MXene-based sensors achieve high selectivity, rapid responses, and ultralow detection limits for NH_3_ across a room-to-low-temperature operational range. Their successful deployment in complex scenarios, including industrial safety monitoring, food quality control, agricultural/livestock management, and medical diagnostics, validates the significant potential for transitioning this technology from laboratory research to practical implementation. Future research should focus on improving the antioxidant properties, cross-sensitivity, and moisture resistance of materials, and on integrating them with machine learning algorithms to provide core support for the construction of real-time, accurate, and low-power environmental and health monitoring networks.

## Figures and Tables

**Table 1:** Different MXene-based sensors for detecting ammonia gas.

Composite Material Type	Material	Sensitivity	Work Temperature	Res/Rec (s)	Detection Limit	Reference
Metal oxides	CeO_2_/Nb_2_CT_x_	51.2% (50 ppm)	25 °C	70/298	500 ppb	[33]
	In_2_O_3_/Ti_3_C_2_T_x_	5 (50 ppm)	25 °C	60/300	1 ppm	[36]
	Ti_3_C_2_T_x_/TiO_2_	4.7% (10 ppb)	31 °C	33/277	500 ppb	[38]
	Ti_3_C_2_Tx MXene/GO/CuO/ZnO	59.9% (100 ppm)	Room temperature	26/25	25 ppm	[24]
	*α*-Fe_2_O_3_/Ti_3_C_2_T_x_	18.3% (5 ppm)	Room temperature	2.5/2	5 ppm	[69]
Polymers	PANI/Ti_3_C_2_T_x_	55.9% (20 ppm)	25 °C	Feb-50	5 ppm	[47]
	PEDOT:PSS/Ti_3_C_2_T_x_	20% (25 ppm)	20 °C	280/393	10 ppm	[56]
	Ti_3_C_2_T_x_/TiO_2_/PPy	28% (5 ppm)	25 °C	162/260	5 ppm	[59]
	MXene/MoS_2_/PPy	21% (100 ppm)	Room temperature	33/277	10 ppm	[70]
	PPy/Ti_3_C_2_T_x_	26% (100 ppm)	Room temperature	62/451	5 ppm	[71]
Two-dimensional materials	Nb_2_CT_x_/MoSe_2_	71% (50 ppm)	25 °C	15/20	1 ppm	[41]
	MoS_2_/Ti_3_C_2_	10% (100 ppm)	Room temperature	7-Oct	1 ppm	[43]
	ReS_2_/Ti_3_C_2_T_x_	7.8% (10 ppm)	25 °C	40/50	1 ppm	[46]
	MXene/SnS_2_	42.9% (10 ppm)	Room temperature	161/80	10 ppb	[72]
	WS_2_/MXene	15.5% (5 ppm)	25 °C	160/100	100 ppb	[73]
Precious metals	Ag@Ti_3_C_2_T_x_	64.07% (100 ppm)	25 °C	230/172	10 ppm	[61]
	Ti_3_C_2_T_x_/TiO_2_/Ru	15.06% (100 ppm)	Room temperature	113/381	5 ppm	[63]
	Au/Ti_3_C_2_T_x_ and Pt/MXene	16% and 9% (50 ppm)	25 °C	190/650	10 ppm	[42]
	Au/*α*-Fe_2_O_3_/Ti_3_C_2_T_x_	16.9% (1 ppm)	Room temperature	2-Mar	1 ppm	[74]
	Pt@SnS_2_/Ti_3_C_2_T_x_	22.7 (10 ppm)	Room temperature	164/38	23 ppb	[75]
